# Long non-coding RNA TUG1 as a potential prognostic biomarker in human cancers: a meta-analysis

**DOI:** 10.18632/oncotarget.19099

**Published:** 2017-07-08

**Authors:** Peng-Ju Ma, Qing-Kai Guan, Lei Meng, Nan Qin, Jia Zhao, Bao-Zhe Jin

**Affiliations:** ^1^ Department of Neurosurgery, The First Affiliated Hospital of Xinxiang Medical University, Xinxiang 453000, Henan Province, People’s Republic of China

**Keywords:** TUG1, neoplasms, prognosis, metastasis, meta-analysis

## Abstract

LncRNA taurine upregulated gene 1 (TUG1) is reportedly dysregulated in various cancers. We performed this meta-analysis to clarify the usefulness of TUG1 as a prognostic marker in malignant tumors. The PubMed, Medline, OVID, Cochrane Library, and Web of Science databases were searched from inception to Jan 11, 2017. Hazard ratios (HRs) and 95% confidence intervals (CIs) were calculated to explore the relationship between TUG1 expression and overall survival (OS). Odds ratios (ORs) were calculated to assess the association between TUG1 expression and pathological parameters. Thirteen original studies covering 1,274 cancer patients were included in this meta-analysis. The pooled HR suggested that high TUG1 expression correlated with poor OS (pooled HR=1.41, 95% CI: 1.01-1.98) in cancer types other than non-small cell lung cancer. TUG1 expression was also related to distant metastasis (OR=3.24, 95% CI: 1.18-8.93), large tumor size (OR=4.07, 95% CI: 1.08-15.28) and advanced tumor stage (OR=3.45, 95% CI: 2.19-5.44). Begg’s funnel plot and Egger’s test showed no evidence of obvious asymmetry for overall survival or tumor stage. Thus high TUG1 expression appears predictive of poor OS, distant metastasis, advanced tumor stage and large tumor size. This suggests TUG1 expression could serve as a biomarker for poor prognosis in cancers.

## INTRODUCTION

According to the American Cancer Society, approximately 1.7 million new cancer cases and 600 thousand cancer deaths are projected to occur in American in 2017 [1]. Furthermore, it has been reported that 8.2 million people die from cancers and 14.1 million people are diagnosed with cancer in 2012 worldwide [2]. Despite recent advances in clinical treatment, cancer continues to be a leading cause of death worldwide, owing to delayed diagnosis, poor prognosis, recurrence and development of resistance by cancer cells. Therefore, efforts to develop new prognostic markers should be made to help modify clinical application in cancers.

Long noncoding RNAs (lncRNAs) are non-protein-coding RNA molecules longer than 200 nucleotides. According to the Encyclopedia of DNA Elements (ENCODE) project, the transcripts cover 62-75% of our genome, among which are mostly noncoding RNAs [3]. LncRNAs have many important functions in disease, including epigenetic regulation and transcriptional and posttranscriptional regulation [4]. Recently, dysregulation of lncRNAs has bee reported in various types of cancer [5–8]. Some lncRNAs play a vital role in cancer progression, affecting proliferation, invasion and metastasis [9–10]. This suggests LncRNAs may be a useful marker of cancer prognosis and metastasis [11].

In 2005, lncRNA Taurine Upregulated Gene 1 (TUG1) was initially identified as a transcript up-regulated by taurine in mouse retinal cells [12]. TUG1 is located at chromosome 22q12. Recently, more and more scientists have found that TUG1 might play important roles in cancer proliferation and metastasis, and TUG1 expression may have a relationship with prognosis and metastasis of human cancers [13]. However, most studies reported so far are limited in discrete outcome and sample size. Up to date, no meta-analysis has been performed to examine the relationship between TUG1 and the relevant clinical outcomes as well as clinicopathological parameters and survival. Therefore, we performed this meta-analysis to investigate whether TUG1 could serve as a molecular marker for prognosis prediction in human cancers.

## RESULTS

### Study characteristics

**Figure 1:**
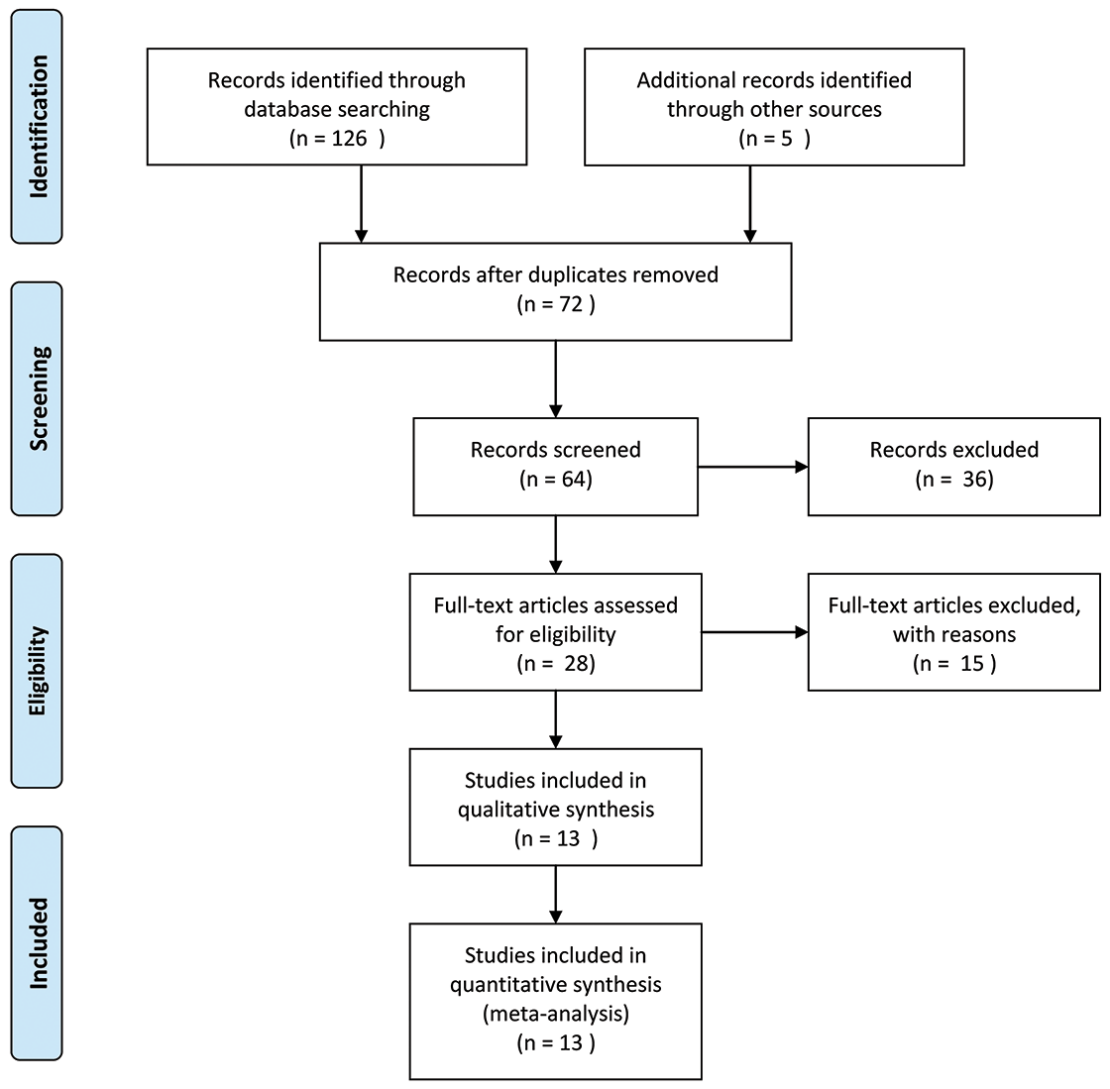
Flowchart showing the steps of literature search and selection criteria for the meta-analysis

The detailed screening process is shown in detail in Figure 1. According to the inclusion and exclusion criteria, thirteen studies and 1,274 patients were included in the meta-analysis [14–26]. Additionally, the characteristics of the 13 studies included in the present meta-analysis are summarized in Table 1. The subject number of 13 studies ranged from 33 to 218, with a mean sample size of 98. Twelve of the thirteen studies were conducted in China whereas one study was from Czech Republic and were published between 2014 and 2017. Among the thirteen studies, two focused on non-small cell lung cancer [20, 24], and one each on hepatocellular carcinoma [14], muscle-invasive bladder cancer [15], esophageal squamous cell carcinoma [16], ovarian cancer [17], glioma [18], breast cancer [19], osteosarcoma [21], small cell lung cancer [22], colorectal cancer [23], renal cell carcinoma [25], gastric cancer [26]. TUG1 expression was measured in cancerous specimens. All of the diagnoses of DM, tumor size and tumor stage were all dependent on the pathology. The reference gene of TUG1 in these studies were found to be inconsistent, including GAPDH [14, 16–25], RNU48 [15] and β-actin [26]. The Newcastle-Ottawa Scale (NOS) confirmed that all studies were of good quality (Table 2).

**Table 1:** The basic information and data of all included studies in the meta-analysis

Study	Year	Region	Tumor type	Sample size	TUG1 expression						Analysis (OS)	HR(95% CI) high / low	Reference gene	NOS	Method
					High			Low							
					Total	DM	HS	Total	DM	HS					
Huang^[14]^	2015	China	HCC	77	47	-	33	30	-	6	-	-	GAPDH	7	PCR
Iliev^[15]^	2016	Czech	MIBC	47	26	-	-	21	-	-	Multivariate	2.54(1.13-5.74)	RNU48	7	PCR
Jiang^[26]^	2016	China	ESCC	218	109	-	64	109	-	50	Multivariate	1.403(1.012-1.946)	GAPDH	8	PCR
Kuang ^[17]^	2016	China	OC	62	33	-	25	29	-	12	-	-	GAPDH	7	PCR
Li^[18]^	2016a	China	Glioma	120	60	-	-	60	-	-	Multivariate	0.57(0.34-0.96)	GAPDH	7	PCR
Li^[19]^	2016b	China	BC	100	55	34	44	45	8	16	-	-	GAPDH	7	PCR
Lin^[20]^	2016	China	NSCLC	89	31	-	-	58	-	-	Multivariate	0.77(0.27-2.22)	GAPDH	7	PCR
Ma^[21]^	2015	China	OSC	76	41	-	9	35	-	3	Multivariate	2.78(1.29-6.00)	GAPDH	8	PCR
Niu^[22]^	2017	China	SCLC	33	16	-	12	17	-	5	Multivariate	1.61(0.52-4.99)	GAPDH	7	PCR
Sun^[23]^	2016	China	CRC	120	71	18	36	49	7	15	Multivariate	2.15(1.29-3.58)	GAPDH	8	PCR
Zhang^[24]^	2014	China	NSCLC	192	96	-	-	96	-	-	Multivariate	0.46(0.31-0.68)	GAPDH	7	PCR
Zhang^[25]^	2016a	China	RCC	40	31	-	9	9	-	2	-	-	GAPDH	7	PCR
Zhang^[26]^	2016b	China	GC	100	50	3	28	50	2	15	Multivariate	1.066(1.023-1.112)	β-actin	8	PCR

**Note:** The dashes represent no data.

HCC, hepatocellular carcinoma; MIBC, muscle-invasive bladder cancer; ESCC, esophageal squamous cell carcinoma; OC, ovarian cancer; BC, breast cancer; NSCLC, non-small cell lung cancer; OSC, osteosarcoma; SCLC, small cell lung cancer; CRC, colorectal cancer; RCC, renal cell carcinoma; GC, gastric cancer; DM, distant metastasis; HS, high stage; OS, overall survival; HR, hazard ratio; NOS, Newcastle-Ottawa Scale.

**Table 2:** Study quality was assessed according to the Newcastle-Ottawa Scale

Author	Country	Adequate of case definition	Representativeness of the cases	Selection of controls	Definition of controls	Comparability of cases and controls	Ascertainment of exposure	Same method of ascertainment	Non-response rate
Huang^[14]^	China	★	★	★	NA	★★	★	★	NA
Iliev^[15]^	Czech	★	★	★	NA	★★	★	★	NA
Jiang^[26]^	China	★	★	★	★	★★	★	★	NA
Kuang ^[17]^	China	★	★	★	NA	★★	★	★	NA
Li^[18]^	China	★	★	★	NA	★★	★	★	NA
Li^[19]^	China	★	★	★	NA	★★	★	★	NA
Lin^[20]^	China	★	★	★	NA	★★	★	★	NA
Ma^[21]^	China	★	★	★	NA	★★	★	★	NA
Niu^[22]^	China	★	★	★	NA	★★	★	★	NA
Sun^[23]^	China	★	★	★	NA	★★	★	★	NA
Zhang^[24]^	China	★	★	★	NA	★★	★	★	NA
Zhang^[25]^	China	★	★	★	NA	★★	★	★	NA
Zhang^[26]^	China	★	★	★	★	★★	★	★	NA

Notes: The quality of the article is through the number of stars(*). Through the selection and exposure categories, each numbered item gets a star at most. A maximum of two stars can be given for comparability. NA: not available

### Association between the TUG1 expression level and OS

**Figure 2:**
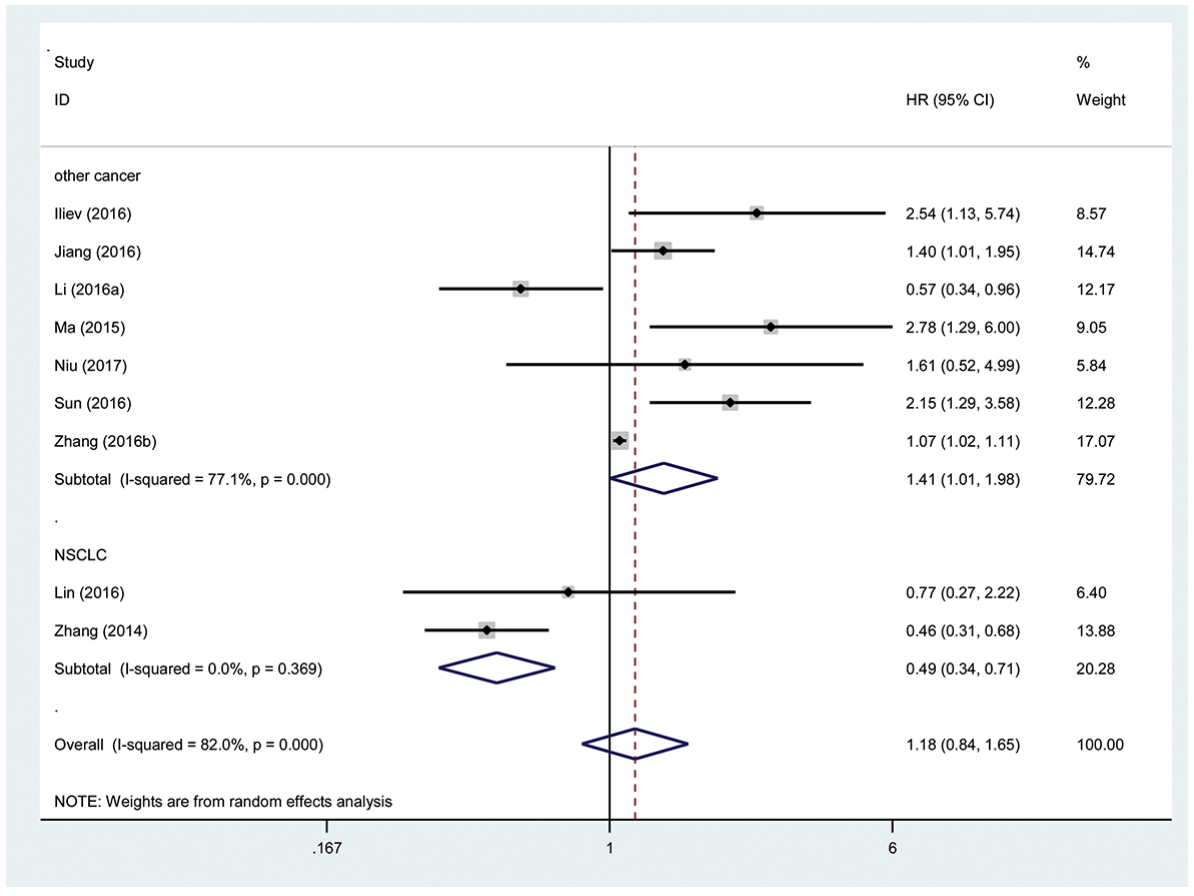
Forest plot showing association between OS and elevated TUG1 expression in the different types of cancer

We performed a cumulative meta-analysis to assess the function of TUG1 for overall survival (OS) in patients with cancer. Additionally, nine included studies with 995 patients reported the relationship between OS and TUG1. The random effects model was used due to significant heterogeneity (I^2^=82%, P_Q_=0.000). Due to the presence of heterogeneity, subgroups were analyzed based on cancer type (NSCLC or other cancer). A significant association was observed between TUG1 and OS in other cancer patients (pooled HR=1.41, 95% CI: 1.01-1.98; Figure 2). Furthermore, the subgroups were analyzed based on the cancer type and revealed a significant association between TUG1 and OS in NSCLC (HR=0.49, 95% CI: 0.34-0.71).

This result demonstrated that a high expression of TUG1 might be correlated with a shorter OS in other types of cancer that excluded NSCLC. Thus, we found that TUG1 was an independent factor of OS among patients with cancer.

### Association between the TUG1 expression level and DM

**Figure 3:**
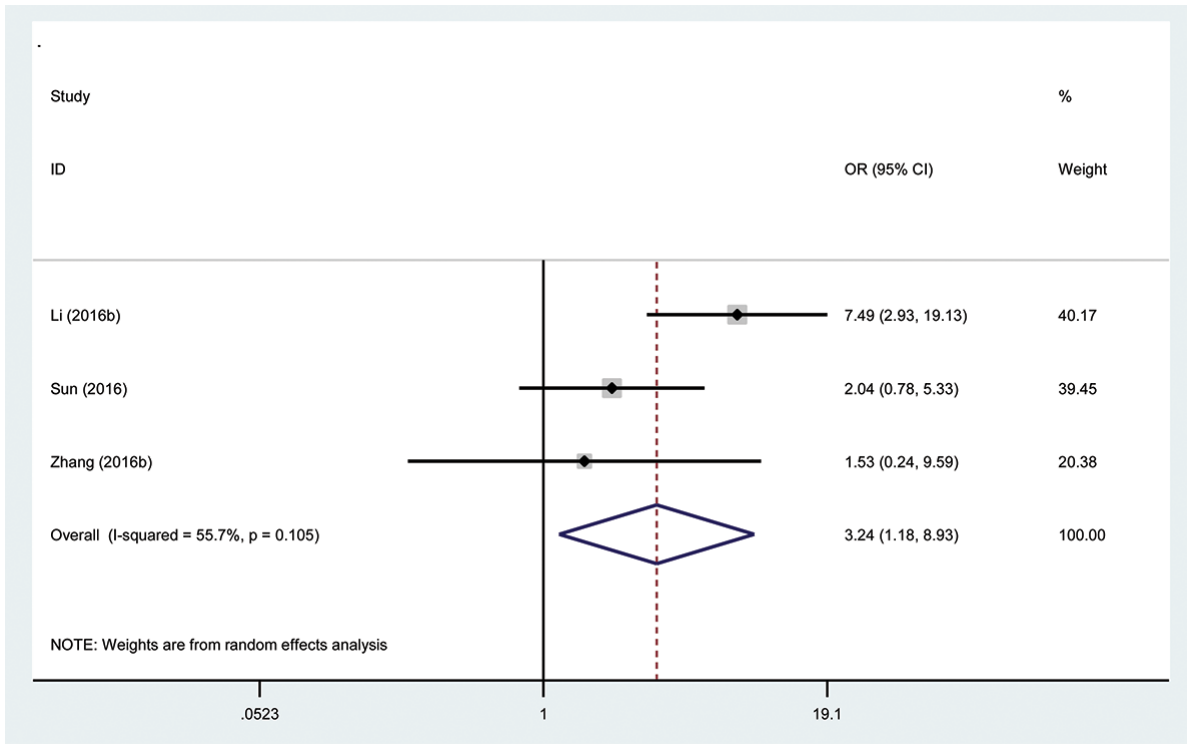
Forest plot showing association between TUG1 expression levels and distant metastasis

Three hundred twenty patients with cancer from 3 eligible studies were collected and analyzed. The random effects model was used for significant heterogeneity (I^2^=55.7%, P_Q_ =0.105). The odds ratio (OR), expressed as the high TUG1 expression group versus low TUG1 expression group was 3.24 (95% CI: 1.18-8.93, P=0.02; Figure 3). According to the result, there was a significant difference between the two groups in the DM incidence. Additionally, the results demonstrated that a high expression of TUG1 significantly predicted a higher tendency to develop DM in patients with cancer.

### Association between the TUG1 expression level and tumor size

**Figure 4:**
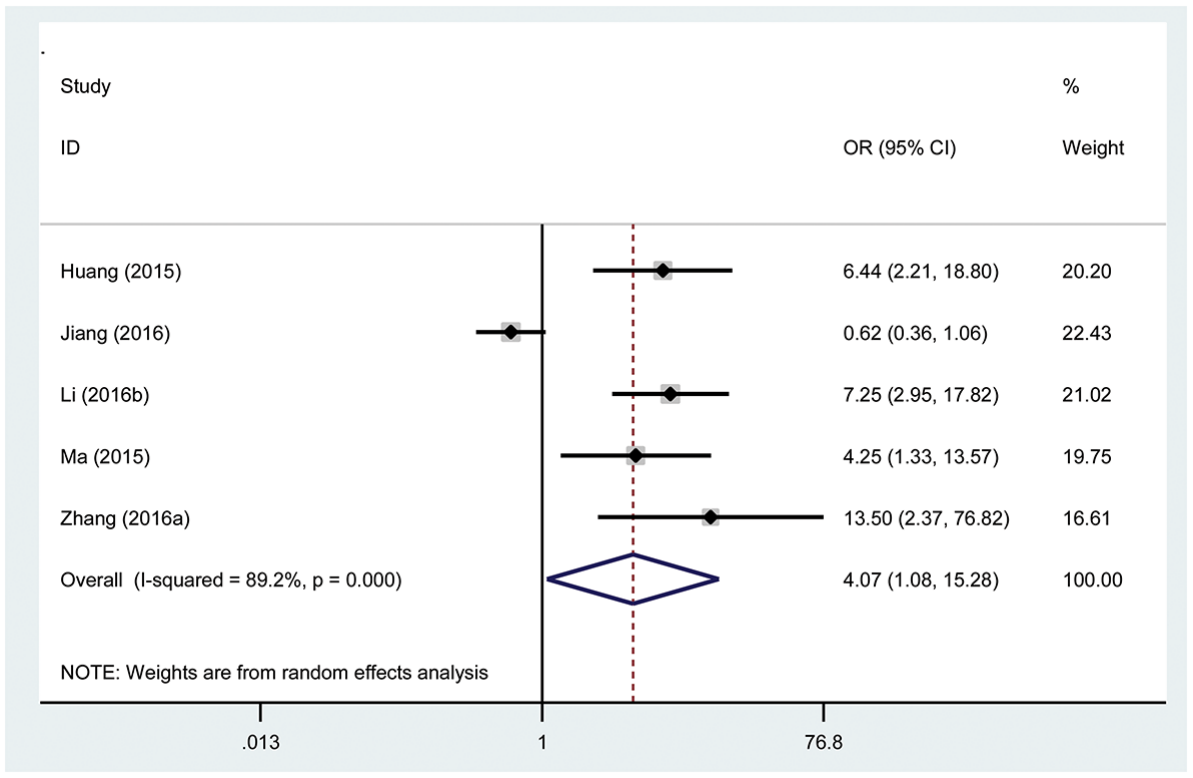
Forest plot showing association between TUG1 expression levels and tumor size

The correlations between TUG1 expression and tumor size are presented in Figure 4. Five studies with 511 patients declared the association between the TUG1 expression levels and number of cancer patients with lager tumor size. There was significant heterogeneity in these studies, and the random-effects model was used (I^2^=89.2%, P_Q_=0.000). The analysis showed a pooled OR =4.07 (95% CI: 1.08-15.28, P=0.04; high versus low TUG1 expression; Figure 4). As a result, the patients with lager tumor size were significantly increased in the high TUG1 expression group. The result revealed that patients with a high TUG1 expression level in tumor tissues may indicate an increased probability of lager tumor size.

### Association between the TUG1 expression level and tumor stage

**Figure 5:**
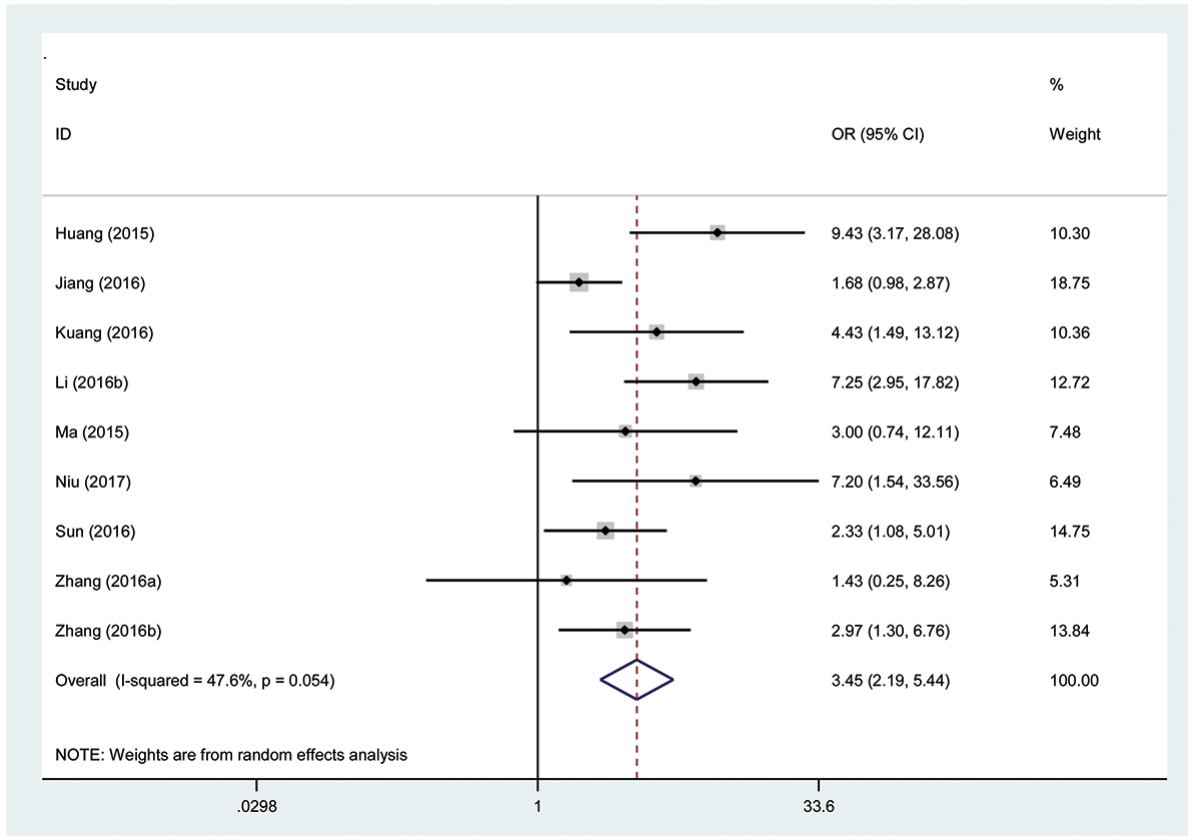
Forest plot showing association between TUG1 expression levels and tumor stage

Eight hundred twenty-six patients in nine eligible studies were included to detect the relationship between the TUG1 expression levels and tumor stage in this meta-analysis. The random effects model was used for significant heterogeneity (I^2^=47.6%, P_Q_=0.054). A significant connection was found between a high TUG1 expression level and high tumor stage in cancer patients (pooled OR= 3.45, 95% CI: 2.19-5.44, P<0.00001; Figure 5).

From the analysis results, the tumor stage was significantly increased in the high TUG1 expression group compared with that in the low TUG1 expression group, and the results demonstrated that a high expression of TUG1 significantly increased the risk of high tumor stage.

### Publication bias

**Figure 6:**
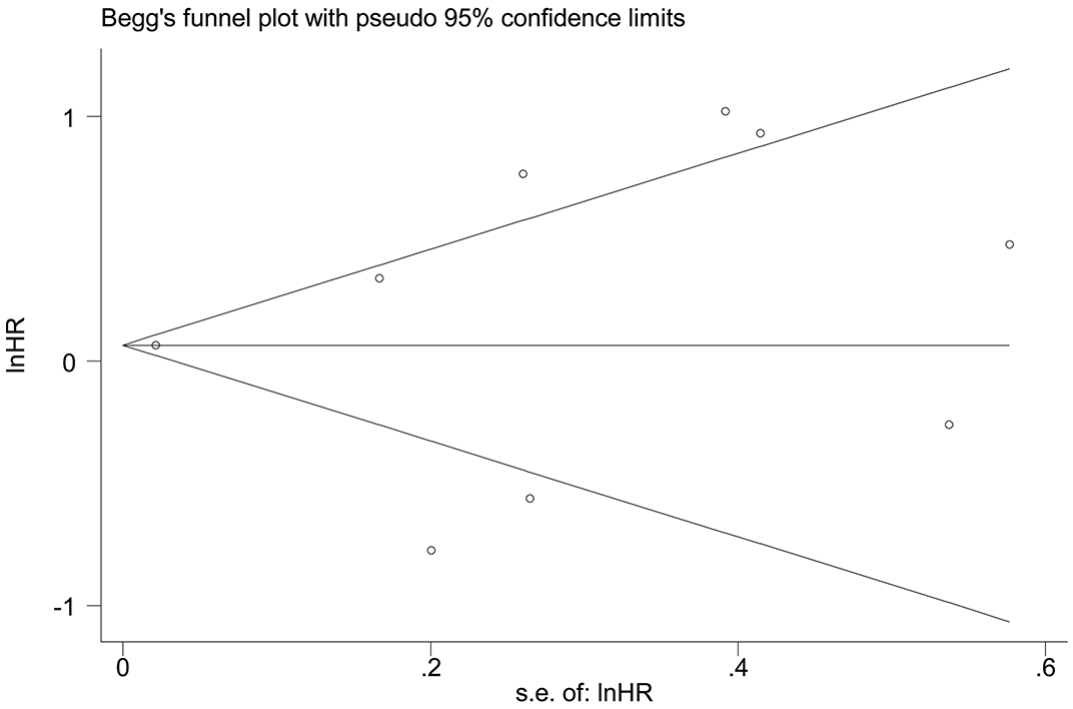
Funnel plot analysis to determine publication bias for the independent role of TUG1 on OS in the different types of cancers

**Figure 7:**
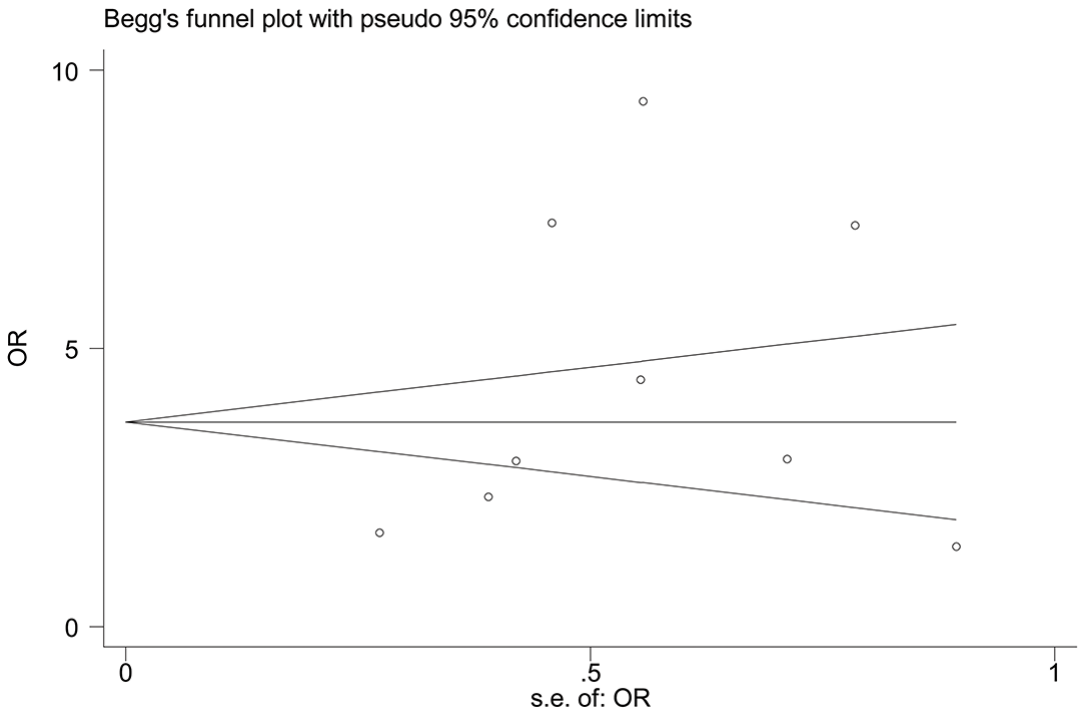
Funnel plot analysis to determine publication bias for the independent role of TUG1 on tumor stage in the different types of cancers

Next, Begg’s funnel plot and Egger’s test were constructed to evaluate publication bias. The results showed no evidence of obvious asymmetry for overall survival (*P*>|t|=0.722, Figure 6). Similarly, there was no evidence for significant publication bias in terms of tumor stage (*P*>|t|=0.192, Figure 7).

## DISCUSSION

Cancer remains a serious threat to human health, and the incidence of cancer has increased gradually in recent years [2]. Most cancers can eventually progress to metastasis. The occurrence of metastasis is an important indicator of a poor prognosis [27, 28]. Moreover, DM has important significance for TNM (tumor–node–metastasis) staging. The precise mechanism underlying metastasis remains uncertain. Cancer research hotspot-molecular biomarkers play a critical role in the prediction and treatment of cancer [29, 30]. It is therefore still necessary and significant to identify new molecular markers to predict tumor metastasis and prognosis.

Recently, genome-wide studies have shown that 80% of transcription of the mammalian genome is associated with lncRNAs [31], and that lncRNAs play a central role in the regulation of differentiation, cell development and proliferation [32]. Moreover, thanks to the specificity of lncRNA expression during the occurrence and progression of tumors, and the ease with which they can be collected from body fluids and tumor tissues, lncRNAs have the potential to serve as useful biomarkers for the diagnosis and monitoring of tumors [33].

Recent studies have shown that TUG1 plays oncogenic roles during tumorigenesis, and that TUG1 is dysregulated in many tumors, including HCC, MIBC, ESCC, OC, BC, NSCLC, OSC, SCLC, CRC, RCC and GC [14–26]. In a previous study, Xie et al. found that TUG1 promoted tumorigenesis by inhibiting POU2F1 expression [34]. Wang and others found that the TUG1 was upregulated in CRC, and they further showed that TUG1 knockdown significantly inhibited cell proliferation, migration and invasion of CRC cells *in vitro* [35]. TUG1 is also a potential oncogene in ESCC that contributes to ESCC cell proliferation and migration [36]. By contrast, TUG1 is downregulated in NSCLC, and high TUG1 expression reportedly correlates with a better prognosis in patients with NSCLC [20, 24]. These studies suggest TUG1 has prognostic value in cancer patients.

Because the utility of TUG1 as a molecular biomarker in human cancer was unclear and contradictory, we conducted this meta-analysis to explore the prognostic value of TUG1 in cancer patients.

One thousand two hundred and seventy-four patients with cancer from 13 eligible studies were collected and analyzed in this study. A random-effects model or fixed-effects model was used depending on the results of heterogeneity analysis. We found that high TUG1 expression may indicate a worse prognosis in cancer patients. By combining HRs from Cox multivariate analyses, there was a significant difference in OS between the high and low TUG1 expression level groups of cancers except NSCLC. Furthermore, statistical analyses revealed that high TUG1 expression in tumor tissues was signiﬁcantly correlated with DM, advanced tumor stage and large tumor size.

Nevertheless, several limitations must be considered while interpreting the conclusions of the present meta-analysis. First, all but one of the included studies were from China (one study was from Czech Republic); consequently, our data may not be globally representative. Second, the included types and numbers of cancers were small. Third, the criteria for high TUG1 expression differed among the included studies. Therefore, additional well-designed and high-quality studies will be needed to confirm these preliminary findings.

## CONCLUSION

In multiple cancers, high levels of TUG1 expression are signiﬁcantly correlated with poor OS, DM, large tumor size and advanced tumor stage. TUG1 expression may thus serve as a promising biomarker for predicting prognosis in cancer patients.

## MATERIALS AND METHODS

### Literature collection

According to the standard guidelines of meta-analyses [37, 38], a systematic search was performed by two authors independently in the electronic databases of Medline, Pubmed, OVID, and Web of Science for relevant articles that concerned TUG1 as a prognostic biomarker for the survival of cancer patients. The latest search was updated on Jan 11, 2017. We performed literature search by both text word and MeSH strategy with the terms ‘‘TUG1’’, “Taurine up-regulated gene 1”, ‘‘lncRNA-TUG1”, ‘‘lncRNA” or ‘‘ noncoding RNA’’ or ‘‘long intergenic noncoding RNA”, ‘‘carcinoma” or ‘‘neoplasm’’ or ‘‘tumor” or ‘‘cancer”, ‘‘prognostic” or ‘‘prognosis’’, ‘‘outcome” or ‘‘survival or ‘‘recurrence’’. The strategy was correspondingly adjusted in the different databases. In the retrieval process, we made a manual search using the reference lists of the relevant articles to include eligible studies.

### Study selection

Two researchers evaluated all of the included studies and extracted the data independently. The inclusion criteria were as follows: 1) the relationship between TUG1 expression and survival was measured in multiple human tumors; 2) the expression levels of TUG1 in human tumor tissue were measured, and the patients were grouped according to the expression levels of TUG1; 3) all of the tumors were conﬁrmed by pathological or histological examinations; 4) studies statistically analyzed patient overall survival or pathological parameters such as DM, tumor size and tumor stage, with respect to TUG1 expression.

The following studies were excluded: 1) reviews, letters, editorials, case reports and expert opinions; 2) non-English language and non-human studies; 3) studies without available data; and 4) laboratory studies with the molecular structure and functions of TUG1 only.

### Data extraction

Two reviewers independently extracted and examined the data from the original articles. Disagreements in the literature assessment were resolved through consensus with a third reviewer. The following data were collected: surname of the first author, publication year, country, tumor type, sample size, the number of patients with distant metastasis and high tumor stage, HR and 95% CI of elevated TUG1 for OS, reference gene of TUG1, the NOS score, and detection method of TUG1.

The study quality was assessed in accordance with the Newcastle-Ottawa Scale (NOS). Nine items were extracted, and each item scored 1. The total scores ranged from 0 to 9. If the scores were ≥7, the study was considered as high quality.

### Statistical methods

Statistical analyses were performed using Stata version 12.0 software. The heterogeneity among different studies was measured by the Q and I^2^ tests. A probability value of I^2^ ≥ 50%, and P < 0.1 indicated the existence of significant heterogeneity [39]. A random effects model or fixed effects model was used depending on the results of heterogeneity analysis. If there was a significant heterogeneity among the studies, the random-effects model was adopted. The potential publication bias was assessed by the Begg’s funnel plot and Egger’s test. Pooled HRs and ORs were extracted from the published data. If the HRs can be obtained directly from the publication, we used crude ones. While the HR and 95% CI were not directly reported in the studies, survival information was extracted from Kaplan-Meier curves and was used to estimate the HR. The log HR and SE were used to summarize the outcome of overall survival [40]. OR and their 95% CI were combined to assess the association between TUG1 expression and clinicopathological parameters, including DM, tumor size and tumor stage.
